# Ancient Polyploidy and Genome Evolution in Palms

**DOI:** 10.1093/gbe/evz092

**Published:** 2019-04-27

**Authors:** Craig F Barrett, Michael R McKain, Brandon T Sinn, Xue-Jun Ge, Yuqu Zhang, Alexandre Antonelli, Christine D Bacon

**Affiliations:** 1 Department of Biology, West Virginia University; 2 Department of Biological Sciences, University of Alabama; 3 Key Laboratory of Plant Resources Conservation and Sustainable Utilization, South China Botanical Garden, Chinese Academy of Sciences, Guangzhou, PR China; 4 Department of Biological and Environmental Sciences, University of Gothenburg, Sweden; 5 Gothenburg Global Biodiversity Centre, Göteborg, Sweden; 6 Royal Botanical Gardens Kew, Richmond, United Kingdom

**Keywords:** Arecaceae, Arecales, chromosome, dysploidy, genome duplication, genome size

## Abstract

Mechanisms of genome evolution are fundamental to our understanding of adaptation and the generation and maintenance of biodiversity, yet genome dynamics are still poorly characterized in many clades. Strong correlations between variation in genomic attributes and species diversity across the plant tree of life suggest that polyploidy or other mechanisms of genome size change confer selective advantages due to the introduction of genomic novelty. Palms (order Arecales, family Arecaceae) are diverse, widespread, and dominant in tropical ecosystems, yet little is known about genome evolution in this ecologically and economically important clade. Here, we take a phylogenetic comparative approach to investigate palm genome dynamics using genomic and transcriptomic data in combination with a recent, densely sampled, phylogenetic tree. We find conclusive evidence of a paleopolyploid event shared by the ancestor of palms but not with the sister clade, Dasypogonales. We find evidence of incremental chromosome number change in the palms as opposed to one of recurrent polyploidy. We find strong phylogenetic signal in chromosome number, but no signal in genome size, and further no correlation between the two when correcting for phylogenetic relationships. Palms thus add to a growing number of diverse, ecologically successful clades with evidence of whole-genome duplication, sister to a species-poor clade with no evidence of such an event. Disentangling the causes of genome size variation in palms moves us closer to understanding the genomic conditions facilitating adaptive radiation and ecological dominance in an evolutionarily successful, emblematic tropical clade.

## Introduction

Genomic studies across the eukaryotic tree of life reveal that genome size is not indicative of organismal complexity, known as the “C-value paradox,” or “C-value enigma” (e.g., Thomas 1971; Cavalier-Smith 1978; Lewin 1983; Gregory 2001, 2005). For example, the genomes of some simple chlorophyte algae are orders of magnitude larger than the genomes of many flowering plants, despite multicellularity and the extensive differentiation of tissues found in the latter. Genome size and complexity have been hypothesized to correlate with or even drive rates of speciation, but evidence is equivocal (reviewed by Kraaijeveld [2010]). Instead, polyploidy and genome size variation may be more strongly correlated with species richness among major plant clades (e.g., Soltis et al. 2009; Wood et al. 2009; Jiao et al. 2011; Puttick et al. 2015). Plant genomes vary immensely in size (2,400-fold), from 61 megabases (Mb) in the carnivorous *Genlisea* (Fleischmann et al. 2014) to the lilioid species *Paris japonica*, at 148.8 gigabases (Gb; Pellicer et al. 2010).

What causes such drastic genome size variation in plants? Genome expansion in plants occurs by well-characterized mechanisms, and polyploidy, including both autopolyploidy and allopolyploidy, is often implicated (e.g., Hawkins et al. 2008; Soltis et al. 2009; Grover and Wendel 2010; Wendel 2015; Kellogg 2016). Genome expansion may also occur via tandem or segmental duplication of chromosomal regions (e.g., Zhang 2003). Genome size reduction is less well understood. From a mechanistic perspective, genomes can decrease in size via fractionation and diploidization following a polyploidy event, wherein chromosomes undergo purging of many duplicated regions and structural rearrangements; illegitimate recombination between chromosomes, where misalignment during synapsis leads to large chromosomal deletions; intrastrand recombination, where misalignments occur within a single chromosome leading to large deletions; homologous recombination during meiosis; and chromosomal inversions, particularly those that expose formerly pericentric regions to the distal, telomeric ends of chromosomes where they can be more easily be deleted (Devos et al. 2002; Bennetzen et al. 2005; Hawkins et al. 2008; Zenil-Ferguson et al. 2016; Ren et al. 2018). Transposable elements also play a major role in both genomic growth via bursts of, for example, copy-paste transposition, and in genomic downsizing by causing misalignments during synapsis (e.g., Bennetzen et al. 2005).

Monocots, comprising nearly one fourth of all flowering plant species, display the largest range of genome size variation among flowering plants (Leitch et al. 2010). Among monocots, the palms (family Arecaceae, with >2,500 accepted species) represent a diverse and ancient clade >100-Myr old (Couvreur et al. 2011; Givnish et al. 2018) and comprise major ecological components of all tropical ecosystems on Earth, especially in Southeast Asia and the Neotropics, where they are particularly diverse and abundant (Uhl and Dransfield 1987; Dransfield et al. 2008; ter Steege et al. 2013; Baker and Dransfield 2016; Balslev et al. 2016). Palms are of immense economic importance as ornamentals, in oil production, and in many tropical areas such as Amazonia they are nearly as important as members of the grass family for human nutrition and shelter (Cámara-Leret 2014; Baker and Dransfield 2016; Balslev et al. 2016). Palms are divided into five subfamilies: Arecoideae (111 genera/1,390 species), Ceroxyloideae (8/47), Coryphoideae (47/505), Nypoideae (1/1; *Nypa fruticans*); and Calamoideae (21/645) (Asmussen et al. 2006; Dransfield et al. 2008; Baker and Dransfield 2016). Most recent analyses based on complete sets of plastid genes support placement of Arecaceae as sister to a small family, Dasypogonaceae. In contrast to the diverse and pantropical palms, this family contains only four genera and 18 species, and is restricted to Mediterranean habitats of southern and western Australia (Givnish et al. 2010; Barrett et al. 2013, 2016; Givnish et al. 2018). Both families have been placed in the order Arecales (The Angiosperm Phylogeny Group 2016), but recent studies have revealed that these ancient lineages should be recognized as distinct, as together they lack a uniquely definitive synapomorphy and diverged >100 Ma (e.g., Givnish et al. 2018).

The evolutionary dynamics of genomes are poorly understood in the Arecales (sensu stricto, i.e., the palms) and even more so for the Dasypogonales (Leitch et al. 2010). There is a 33-fold range in genome size across the palms, which typically harbor from 2*n* = 26–36 chromosomes, though *Voanioala* is a remarkable outlier with 2*n* = 596 (Johnson et al. 1989; Röser 1997). Leitch et al. (2010) compared genome sizes for each chromosome number class among palms, from 2*n* = 26–36 and concluded that “changes in genome size can occur with no alteration of chromosome number leading to related species having significantly different sized chromosomes.” Evidence for polyploidy in the palms is piecemeal, for example, in the arecoid tribe Cocoseae (Gunn et al. 2015). Instances of allopolyploidy in sympatry may occur more widely, based on putative hybrid introgression in some genera, but detailed genomic studies are lacking to pinpoint causality (e.g., *Attalea*, *Brahea*, *Coccothrinax*, *Copernicia*, *Geonoma*, *Latania*, *Phoenix*, *Pritchardia*, and *Ptychosperma*; Glassman 1999; Dransfield et al. 2008; Ramírez-Rodríguez et al. 2011; Bacon et al. 2012). Observations based on comparing silent substitutions among duplicate gene pairs (Ks plots) suggest at least that oil and date palms (*Elaeis guineensis* and *Phoenix dactylifera*, respectively) show evidence of past whole-genome duplications (WGDs) (Al-Mssallem et al. 2013; Singh et al. 2013). The only formal phylogenomic analyses to include more than one palm species are those of D’Hont et al. (2012) and McKain et al. (2016), providing more conclusive evidence of a shared WGD event among the two model palms, which represent subfamilies Arecoideae and Coryphoideae, respectively.

Several questions remain with respect to genome evolution in the palms. Did WGD events influence genome evolution across the palms and close relatives, and if so, how and at what point in their evolutionary history? Does variation in genome size and chromosome number carry phylogenetic signal across palms and relatives? Here, we use publicly available and newly generated transcriptomic and genomic data, a densely sampled phylogenetic tree, and published data on genome size and chromosome number to address the above questions. Our specific objectives are to 1) reconstruct the evolution of genome size and chromosome number and 2) detect and place the hypothesized WGD event(s), both within a phylogenetic context.

Palms are a model lineage in which to test relationships among trait evolution, biogeography, paleoenvironments, and tropical biodiversity (e.g., Eiserhardt et al. 2011, 2013; Kissling, Baker, et al. 2012; Kissling, Eiserhardt, et al. 2012; Baker and Couvreur 2013; Couvreur and Baker 2013; Bacon et al. 2018). Analyses in palms will help to elucidate patterns of genome size evolution in long-lived monocots, which are typically understudied in the world of evolutionary genomics. Ultimately, our aim is to generate a framework in which to integrate genome evolutionary dynamics, biogeography, and trait evolution to elucidate the drivers of palm biodiversity.

## Materials and Methods

### Phylogenetic Trees

Two recently published trees include dense taxon sampling for the palms (Faurby et al. 2016; Antonelli et al. 2017). The “SUPERSMART” tree (Antonelli et al. 2017) was chosen because it has the best taxonomic representation that matches the available genome size, chromosome number, and genome skim data (see below). The tree contains 733 species and 293 genera and is based on all publicly available data from 37 loci (see Antonelli et al. 2017 for details).

### Transcriptomic Data

Data were compiled from previously published RNA-seq data sets across monocots from the Sequence Read Archive (https://www.ncbi.nlm.nih.gov/sra; last accessed December 12, 2018), and the OneKP Project (https://sites.google.com/a/ualberta.ca/onekp/; last accessed December 12, 2018). Complete genomes were downloaded from GenBank (https://www.ncbi.nlm.nih.gov/genbank; last accessed December 12, 2018). Additional RNA-seq data sets were generated for *Chamaedora seifrizii* (Arecaceae: Arecoideae) and one representative species from four genera in family Dasypogonaceae: *Baxteria australis*, *Calectasia narragara*, *Dasypogon bromeliifolius*, and *Kingia australis* (supplementary table S1, Supplementary Material online).

### Genome Size and Chromosome Numbers

Genome sizes and chromosome numbers were obtained from the Royal Botanic Gardens, Kew Angiosperm DNA C-values database (Bennett and Leitch 2012; http://data.kew.org/cvalues/) and Dransfield et al. (2008), using only “prime” estimates (i.e., excluding those with low confidence). Data and trees were pruned in the “APE” package of R (Paradis and Schliep 2019) to match sampled tips from the SUPERSMART tree at the species level.

### Data and Tree Articulation

We attempted to maximize the match of each data set (tree, chromosome number, and genome size) at the species level (supplementary fig. S1 and table S2, Supplementary Material online). In cases where genome size, chromosome number, or genome skim data did not match at the species level, and there were multiple genome size estimates represented by different species within a genus, we used another species of the same genus for the genome size estimate. Although ideally, we would prefer only data from the same species for genome size (further, even from the same individuals per species), using a congener is unlikely to bias our results, because the focus of this analysis is on large-scale relationships among repeat fractions and genome sizes.

### Transcriptome Assembly and Gene Tree Reconstruction

RNA-seq data were assembled using Trinity v.2.2.0 (Grabherr et al. 2011; Haas et al. 2013) as described in McKain et al. (2016). Reads were cleaned using Trimmomatic v.0.32 (Bogler et al. 2014) with adapter trimming for TruSeq adapter sequence using one seed mismatch, a palindrome threshold of 30, and a simple clip threshold of 10. After adapter trimming, a sliding window of 10 base pairs a minimum threshold average Phred score of 20 was used to trim reads based on quality. Finally, reads <40 bp in length were discarded. Once assembled, reads were mapped back to transcripts using bowtie v.1.0.0 (Langmead et al. 2009), and read abundance per transcript was estimated using RSEM v.1.2.29 (Li and Dewey 2011) using the “align_and_estimate_abundance.pl” script packaged with Trinity. FPKM (fragments per kilobase of exon per million fragments mapped) was estimated for each gene identified by Trinity. The percentage of mapped fragments per isoform was estimated and transcripts with a value of <1% were removed from further analysis. FPKM filtered transcripts were translated using the RefTrans pipline (McKain et al. 2016, https://github.com/mrmckain/RefTrans). Transcripts were aligned to gene models from the *Ananas comosum* v.1.0 (Ming et al. 2015), *Asparagus officinalis* v.1.0 (Harkess et al. 2017), *E.guineensis* v.1.0 (Singh et al. 2013), *Oryza sativa* v.7.0 (Kawahara et al. 2013), *Phalaenopsis equestris* v.1.0 (Cai et al. 2015), and *P.dactylifera* v.1.0 (Al-Mssallem et al. 2013) genomes using BlastX with an *e*-value cutoff of 1.0 × e^−10^ (Camacho et al. 2009). BLAST results were filtered to identify best hits as defined by transcript and gene model pairs with the lowest *e*-value and at least 85% bidirectional overlap. Best hit gene models were used to translate transcripts using GeneWise 2.2.0 (Birney et al. 2004). The longest translation for each transcript were used, and if internal stop codons were identified, they were removed from assemblies.

OrthoFinder v.2.2.1 (Emms and Kelly 2015) under default settings was used to circumscribe putative gene families. Diamond v.0.9.19 (Buchfink et al. 2014) with an *e*-value cut off of 0.001 and the BlastP algorithm was used to align sequences to each other for the initial steps of OrthoFinder. In addition to transcriptomes, gene models from genome sequences for *P.dactylifera* v.1.0, *E.guineensis* v.1.0, *Musa acuminata* v.1.0 (D’Hont et al. 2012), *A.comosum* v.1.0, *O.sativa* v.7.0, *Pha.equestris* v.1.0, and *As.officinalis* v.1.0 were used in gene family estimation. Orthogroups were filtered to remove those with sequences from <12 taxa. Amino acid sequences for each orthogroup were aligned using MAFFT v.7.313 with automatic alignment algorithm selection (Katoh and Standley 2013). Aligned amino acid sequences were used to create a codon alignment of the nucleotide sequences using PAL2NAL v.13 (Suyama et al. 2006) under default paramters. Gene trees were reconstructed using RAxML v.8.2.4 (Stamatakis 2014) under a GTR+gamma evolutionary model and 500 standard bootstrap replicates.

Gene trees and accompanying codon alignments were passed to the perl script clone_reducer (Estep et al. 2014; https://github.com/mrmckain/clone_reducer; last accessed December 12, 2018) to identify putative single copy gene families. This script identifies clades with a bootstrap value of 50 or more that comprise a single species. The longest sequence in this clade is then used to represent the clade as a whole. From these reduced alignments, a set of 1,102 gene families were identified as single copy. It is possible that these are not truly single copy but appear single copy due to the incomplete sampling of the genome by transcriptomes. New gene trees were reconstructed for these reduced alignments as described above. The most likely tree for each of these gene families was used to estimate a coalescence-based species tree using ASTRAL—III v.4.XX (Mirabab et al. 2014) using default parameters. Due to its low total transcripts, *Calectasia grandiflora* was not included in the estimation of this species trees. We placed *Calectasia* in the position identified by Barrett et al. 2016, which had a congruent topology to the estimated relationships presented here.

### Identification and Phylogenetic Placement of WGD Events

After filtering for a minimum number of 12 taxa per tree, a total of 6,242 gene trees were used to identify and phylogenetically place putative WGD events. The software PUG (McKain et al. 2016) was used to identify putative gene duplications that coincide with the topology of the reconstructed coalescence-based species phylogeny. We ran PUG with the “estimate_paralogs” parameter flag, which has PUG identify all possible paralogs in a given gene tree by identifying all possible transcript pairs derived from the same taxon in a single gene tree. Each multilabeled gene tree was rerooted to a non-Arecaceae and Dasypogonaceae outgroup with preference given in the order: *Acorus americanus*, *As.officinalis*, *Pha.equestris*, *Typha latifolia*, *A.comosum*, *Neoregalia carolinae*, *O.sativa*, *Hanguana malayana*, *Costus pulverulentus*, *Musa acuminata*, and *Tradascantia paludosa*. With PUG, each putative paralog pair was queried to identify the most recent common ancestor node in the gene tree. The taxon composition of the subtree identified by the most recent common ancestor node was used to identify the equivalent node in the species tree. A placement of the duplication on the species tree was considered acceptable if taxa above the node match those in the gene tree and at least one species sister to this clade in the species tree was found sister to the equivalent clade in the gene tree. For all gene trees and paralogs, we ran PUG to identify both unique duplications (the default) and all duplications (flag “all_pairs”) to identify support for putative WGD events.

### Ancestral State Reconstruction, Shifts, and Phylogenetic Signal

We reconstructed ancestral genome sizes and chromosome numbers initially in the “APE” and “PHYTOOLS” (“contmap” function, Revell 2012) under a Brownian Motion Model. We further applied an Ornstein–Uhlenbeck model to investigate evidence of significant shifts in trait values over time and across the tree using the R package “l1ou” (Khabbazian et al. 2016), which requires no a priori assumptions on the locations of trait shifts. We additionally analyzed evolutionary changes in chromosome number across the tree with ChromEvol (Glick and Mayrose 2014). This software compares explicit models of chromosome evolution by parameterizing ascending and descending dysploidy (where the current number of chromosomes, *j* = *i* + 1 or *i* − 1, respectively, where “*i*” represents the ancestral chromosome number); WGD (*j* = 2*i*); demipolyploidy (*j* = 1.5*i*); chromosome number changes involving a “base” haploid chromosome number (*x*); and linear versus constant rates of change, where linear changes in chromosome number are dependent upon the current chromosome number. We removed *Voanioala gerardii* (2*n* = 596) from the analysis because the sampling in that clade is inadequate to reconstruct such a drastic change in chromosome number. We tested ten models of chromosome evolution under the same set of dysploidy parameters as above. We compared the fit of alternative models via the Akaike Information Criterion (Akaike 1974) and Akaike Weights (Wagenmakers and Farrell 2004). We tested for correlation between log-transformed genome size and chromosome number using phylogenetically independent contrasts (Felsenstein 1985; Garland et al. 1992).

## Results

### Evidence of WGD in Palms

**Fig. 1.**
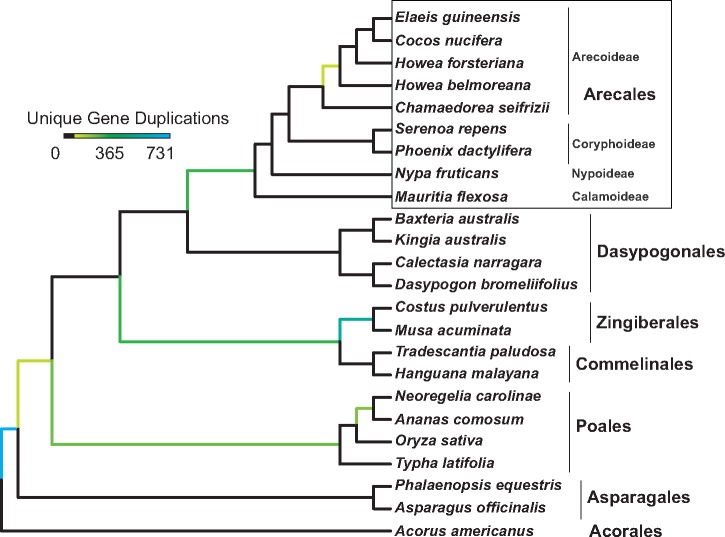
—Phylogenomic evidence for a whole-genome duplication event in the ancestor of all palms. The color bar represents the number of unique gene duplications placed at each node, with at least 80% bootstrap support. The tree has representatives of 4/5 palm subfamilies (Calamoideae = *Mauritia*; Nypoideae = *Nypa*; Coryphoideae = *Phoenix*, *Serenoa*; Arecoideae = *Chamaedorea*, *Cocos*, *Elaeis*, *Howea*).

We found unequivocal evidence for an ancient WGD event shared by all representatives of the palms included here, but not shared with the sister clade, Dasypogonales (fig. 1). Coalescent analysis of relationships based on 1,102 single copy nuclear loci yields a tree with representative Arecoideae sister to Coryphoideae, which together are sister to the monotypic Nypoideae, with *Mauritia*, representing the Calamoideae, the subfamily sister to rest of Arecaceae (fig. 1). Ceroxyloideae were not sampled here. We analyzed a total of 6,242 gene families and detected 2,685 unique gene duplications supporting the species tree topology with a minimal bootstrap value of 80, representing 31.5% of all sampled gene families. The palms shared 278 unique gene duplications (3,321 paralog pairs), representing 4.6% of all gene families analyzed.

### Genome Size

**Fig. 2.**
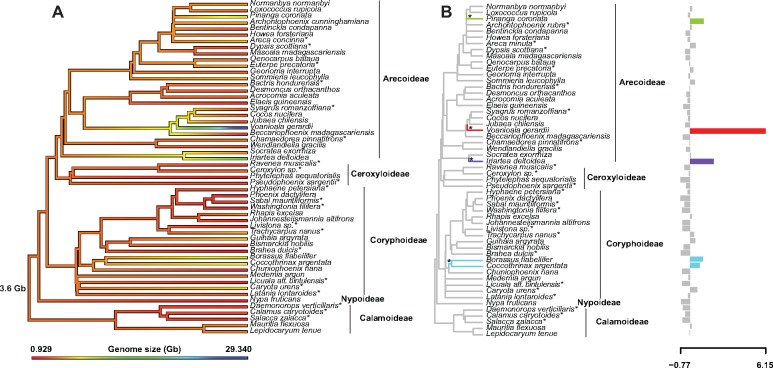
—Ancestral state reconstruction of genome size (in kilobases, kb) in the palms under (*A*) Brownian Motion, where “3.6 Gb” is the ancestral estimate for the palms and (*B*) genome size under an Ornstein–Uhlenbeck model. Asterisks next to species names indicate a different species of the same genus was sampled in the phylogenetic tree; asterisks next to branches in (*B*) represent significant trait shifts (*n* = 4 shifts, BIC = 991.3; Pagel’s *λ* =  7.8 × 10^−5^, *P* = 1.0; Blomberg’s *K* = 0.47, *P* = 0.60).

We found a lack of phylogenetic signal for genome size (fig. 2, *n* = 54 species; Pagel’s *λ* = 7.97 × 10^−10^, *P* = 1.0; Blomberg’s *K* = 0.47, *P* = 0.6). The ancestral genome size for palms under a BM model is ∼3.6 Gb (95% confidence interval, or CI = −0.74 to 8.0 Gb). We found limited evidence for significant shifts in genome size; all of these shifts are increases relative to inferred ancestral values, in *Borassus*, *Coccothrinax*, *Pinanga*, *Iriartea*, and *Voanioala* (fig. 2). Comparison of chromosome number and genome size via phylogenetically independent contrasts yields no significant correlation (*F* = 0.3832, *P* = 0.54).

### Chromosome Number

Ancestral state reconstruction of diploid chromosome number as a continuous character under a BM model yielded a pattern of phylogenetic signal (supplementary fig. S2*A*, Supplementary Material online). The ancestral 2*n* value under BM is 32 for palms (*n* = 195 species). There is a reduction to 2*n* = 26 in *Calamus* (subfamily Calamoideae), and a general increase to 2*n* = 36 in subfamily Coryphoideae. Chromosome number is unchanged at the crown nodes of subfamilies Ceroxyloideae and Arecoideae, and a reduction to 2*n* = 26 is again observed in many species of *Chamaedorea*, for which there is dense sampling relative to other genera. A putative chromosome doubling is observed in *Arenga caudata* relative to all other members of this genus (ancestral 2*n* = 32 → 64), but few other such events are observed in our data set. *Voanioala gerardi*, with 596 chromosomes, was removed as an outlier. We found evidence for 77 shifts in chromosome number across the palms sampled (OU model, BIC = −5,739.041; supplementary fig. S3*B*, Supplementary Material online). We also found significant phylogenetic signal for chromosome number (Pagel’s *λ* = 0.41, *P* = 2.5 × 10^−10^; Blomberg’s *K* = 0.29, *P* = 0.001).

**Fig. 3.**
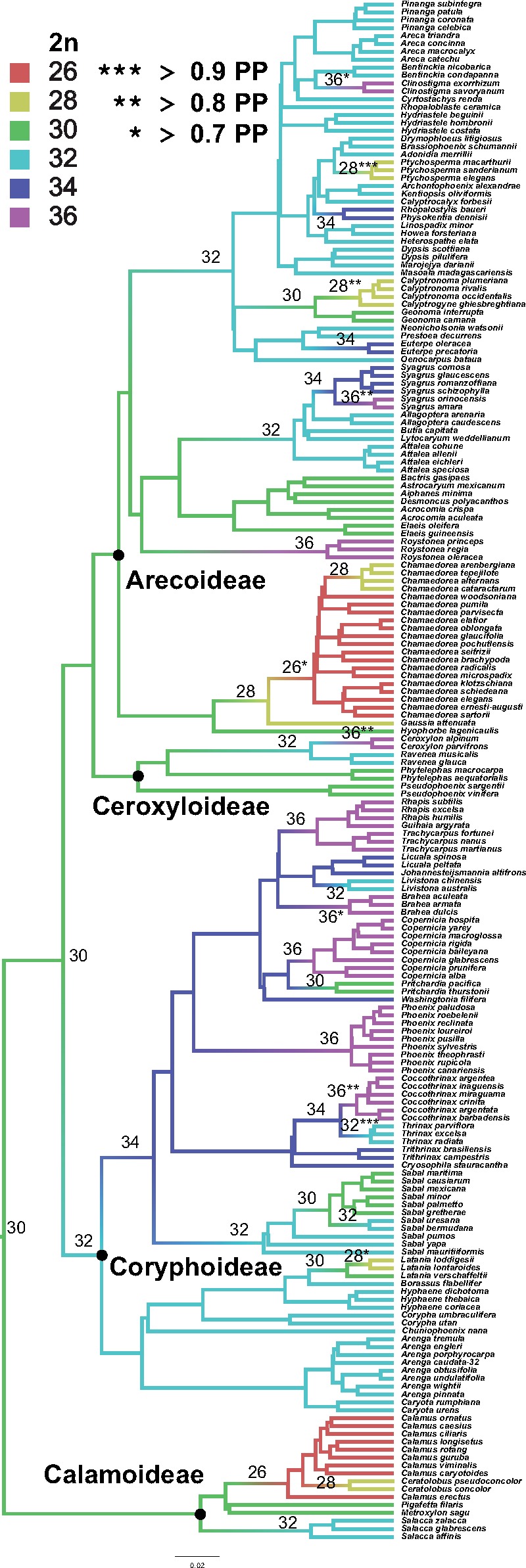
—Maximum-likelihood estimate of ancestral state reconstruction of chromosome number based on a model of linear rate dependency in ChromEvol (i.e., chromosome number changes depend on the current chromosome number), allowing both ascending and descending dysploid changes (*i* + 1, *i* − 1) and WGD (2*i*). Colors correspond to 2*n* chromosome numbers, and numbers in brackets indicate the ML estimate of chromosome number for that node. Asterisks refer to the posterior probability for the highest-likelihood reconstruction of chromosome number. Black dots correspond to four of the five palms subfamilies sampled.

A model of linear dependency had the best fit to our data among ten different models of chromosome evolution in ChromEvol (AIC weight = 0.264; supplementary table S4, Supplementary Material online). The maximum-likelihood estimate for ancestral chromosome number was 2*n* = 30, though posterior probability estimates were low for the deepest nodes of the tree (i.e., PP < 0.7; fig. 3). ChromeEvol detected 34 changes in chromosome number in contrast to the 77 shifts identified under an OU model. Most changes in chromosome number were ascending dysploidy, that is, increases in chromosome number of *n* ≥ 1 (fig. 3*C*), and there was one possible case of WGD in *Arenga caudata* (2*n* = 32 → 64).

## Discussion

Our principal objective was to investigate the evolutionary history of genome evolution in the palms. We found unequivocal evidence of a WGD event shared by all palm subfamilies but not with the sister clade, the monocot order Dasypogonales. We also found evidence of phylogenetic signal for chromosome numbers, evolving predominantly via a linear model of dysploid change.

### Shared WGD in the Palms

We found evidence for a shared WGD event across all palm subfamilies, suggesting that polyploidy likely played a role in the diversification and evolutionary success of this emblematic tropical clade (fig. 1). With our data it may be impossible to infer whether this was the result of auto- versus allo-polyploidy: coupled with extinction, accumulation of substitutions among retained duplicates over long temporal scales has likely saturated any patterns that could be used to distinguish between these two processes. Methods used to detect ancient allopolyploidy mostly center around deep reticulate patterns or preferential paralog retention from one parental species, but all these methods require at least some knowledge of the potential donor lineages (see Clark and Donoghue 2017). The palm WGD event must have occurred between ∼119 and 85 Ma, that is, after the estimated split of orders Arecales and Dasypogonales but before the first divergence of subfamily Calamoideae from the rest of the palms (Couvreur et al. 2011; Givnish et al. 2018). Although previous studies have alluded to a palm WGD, the hypothesis was only based on divergence comparisons of paralogs within genomes and limited taxon sampling (Al-Mssallem et al. 2013; Singh et al. 2013; He et al. 2015; but see D’Hont et al. 2012; McKain et al. 2016 for explicit phylogenomic comparisons). Such “Ks” comparisons, in which frequency distributions of divergence among paralogs are compared within individual genomes or transcriptomes, are informative for evidence of WGD within a particular genome, but they do not provide a rigorous, phylogenetic, comparative test of shared WGD among taxa. In the present analysis, we definitively and precisely confirm the phylogenetic placement of a palm WGD event, moreover indicating that the palm event is older than has been recently hypothesized (70–75 Ma; e.g., van de Peer et al. 2017).

The fact that this WGD event is not shared with the sister clade of palms, order Dasypogonales, is of high significance in terms of potential implications for palm diversification. A growing number of examples like that of Arecales–Dasypogonales is being revealed with the expansion of phylogenomic studies (e.g., see Soltis et al. 2009; Renny-Byfield and Wendel 2014; Panchy et al. 2016). The most comprehensive analysis to date across angiosperms, using RNA-seq data from the 1KP project, revealed that 70 of 99 WGD events are associated with increases in species richness of one clade relative to a species-poor sister clade (Landis et al. 2018). Here, we present a scenario of a species-rich, evolutionarily successful, ecologically dominant, widespread clade with evidence of ancient WGD prior to or coincident with an adaptive radiation. In contrast, its sister clade is relatively species-poor, geographically restricted, ecologically marginal, and lacking evidence of WGD. Although the relationship among ancient WGD and subsequent adaptive radiation (Arecales, vs. a lack thereof in Dasypogonales) may be anecdotal, there are many other diverse plant clades with a history of WGD (van de Peer et al. 2017; Landis et al. 2018). These notably include the order Poales and family Poaceae (Paterson et al. 2009; Tang et al. 2010; McKain et al. 2016), Orchidaceae (Zhang et al. 2017; Unruh et al. 2018), Brassicaceae (Edger et al. 2015), Fabaceae (Lavin et al. 2005; Pfeil et al. 2005; Cannon et al. 2015), and Solanaceae (Schlueter et al. 2004). Thus, it is likely that the ancient WGD identified in this study contributed to palm diversification and ecological dominance in tropical and subtropical ecosystems globally. There is only limited evidence of WGD in palms from the RNA-seq or genome data included in this study (at the base of subfamily Arecoideae, see fig. 1), there are several interesting candidates based on chromosomal information, including, for example, *Arenga*, *Jubaeopsis*, *Rhapis*, and *Voanioala* (Röser et al. 1997; Leitch et al. 2010). It is unclear in *Voanioala* whether repeated rounds of WGD have led to the remarkable proliferation of chromosomes and large genome size, or if another mechanism is responsible, for example, rampant TE accumulation and chromosomal fissions.

Our results naturally prompt a further question: What are the functions of retained paralogs, after post-WGD diploidization has largely purged the duplicated remainder of the genome? We are currently limited in terms of our use of RNA-seq data, as these were taken from a single tissue type (young, developing leaves; e.g., Matasci et al. 2014). Thus, analysis of such a “snapshot” of gene expression may severely limit, or even bias, an assessment of retained duplicate gene function in palms from a whole-organismal perspective. Such an analysis would require more inclusive transcriptomes, sampling multiple tissues both spatially and temporally, as well as complete or draft genomes. This would provide crucial information related to the question of whether WGD did in fact contribute to genetic novelty and thus adaptive radiation in the palms relative to the sister clade, for example, as in the retention of duplicated glucosinolate pathway genes as novel herbivore defense mechanisms in Brassicaceae (Edger et al. 2015).

A second putative palm WGD was found prior to the divergence of the Areceae and Cocoseae tribes in the subfamily Arecoideae (fig. 1). This event is supported by 94 unique gene duplications (285 paralog pairs) with a bootstrap support value of 80% or more. Further investigation is needed to verify this WGD event through increased sampling of Arecoideae, and based on the low support values and the putative paraphyly of *Howea* in the coalescence tree, this may be an artifact. We detected both the *sigma* (228 unique duplication, Bootstrap = 80) and *tau* (731 unique duplications, Bootstrap = 80) events described in earlier analyses (McKain et al. 2016), with *tau* after the divergence of *Acorus*, and sigma prior to the diversification of Poales. We also confirmed previously identified events in Bromeliaceae (196 unique duplications, Bootstrap = 80; McKain et al. 2016), Commelinales + Zingiberales (283 unique duplications Bootstrap = 80, D’Hont et al. 2012), and Zingiberales (538 unique duplications, Bootstrap = 80, D’Hont et al. 2012) (supplementary table S3, Supplementary Material online). There was also signal for a commelinid WGD event occurring after the divergence of Asparagales from the remainder of the monocots but is likely an artifact of sampling.

### Evolution of Genome Size and Chromosome Number

Genome size is not correlated with chromosome number in the palms when accounting for phylogenetic relationships, nor does it carry phylogenetic signal based on our current sampling. Gene space varies among palms, from over 35,000 genes in oil palm to over 40,000 in date palm (Al-Mssallem et al. 2013; Singh et al. 2013). Further, the oil palm genome reveals evidence for a role for segmental gene duplications in gene space expansion (Singh et al. 2013). An estimate based on a recently published transcriptome of *N.fruticans*, a monotypic species of mangrove-growing palms, reveals that up to 45,000 genes may be present (>32,000 were identified via BLAST searches), but these numbers carry great uncertainty as only leaf tissue was sampled (He et al. 2015). Repeat content is known to be a major driver of genome size in plants (e.g., Pellicer et al. 2018). The estimated total repeat content from the date palm genome (transposons, satellite DNA) is ∼38%, whereas this number is greater in oil palm, at an estimated 57% (Al-Mssallem et al. 2013; Singh et al. 2013). It is highly unlikely that increases in gene content alone explain the most drastic examples of genome size expansion in palms (e.g., *Voanioala*), and thus these were likely due to rampant increases in repetitive elements.

Genome size increases appear to be associated with high species diversity in some palm genera but not in others (fig. 2 and supplementary table S2, Supplementary Material online). For example, *Coccothrinax* (up to 7.27 Gb) and *Pinanga* (up to 8.66 Gb) are both relatively species-rich genera (>50 and 100 spp., respectively) with genomes much larger than the ancestral size estimated for palms (3.6 Gb). By contrast, three other genera with large genomes are relatively species poor: (12.01 Gb, one sp., *I. deltoidea*), *Borassus* (8.41, five spp.), and *Voanioala* (38.24 Gb, one sp., *Voanioalagerardii*, but possibly up to four spp.; see Gunn 2004). Clearly more sampling of genome sizes is needed across the palms, especially at—or even below—the species level, allowing a test of the hypothesis that genome size variation and not genome size per se is associated with species diversity (e.g., see Puttick et al. 2015). Ideally, such comprehensive sampling of genome sizes should be paired with phylogenomic information for all species to allow phylogenetically informed comparisons. Moreover, intrageneric and even intraspecific variation in genome size can be substantial (e.g., in *Dypsis*, *Phoenix*, *Pinanga*; summarized in Dransfield et al. [2008] with references therein) necessitating population-level sampling.

We identified major trends in chromosome number evolution across the palms, even with only 195 species sampled for chromosome number and phylogenetic tree information. By explicitly modeling chromosome number across the tree, we detected ∼34 changes in chromosome number, which is fewer than the number of significant shifts detected under an OU model (fig. 3). The treatment of chromosome number as a continuous character may be misleading, and thus explicit models of changes in chromosome number are necessary to effectively capture the evolutionary dynamics of changes across the tree. A linear model of chromosome evolution had the best fit out of ten alternative models (supplementary table S4, Supplementary Material online). This is a state-dependent model in which chromosome number changes are dependent upon the current chromosome number (Glick and Mayrose 2014). Although <10% of the >2,500 palm species were sampled in this study, this suggests that sampling was enough to track a linear mode of evolution across many clades (fig. 3). Large sampling gaps would be expected to obscure the pattern of chromosome number changes; for example, a linear dysploid transition from 2*n* = 30 → 32 → 34 → 36 → 34 → 32 within a lineage or clade might be observed as 2*n* = 30 → 36 → 32 if the taxa with 2*n* = 32, 34, and 34 are not sampled, respectively.

Specifically, ascending dysploidy appears to be the predominant mode of chromosomal change in palms based on the data available, suggesting an overall net trend to more chromosomes. The only information on chromosome number available for the sister clade of palms, Dasypogonaceae, is that of *Dasypogon hookeri*, which contains less than half the ancestral chromosome number of palms (2*n* = 14 vs. 2*n* = 30, fig. 3; Röser 2000; Leitch et al. 2010). It is plausible that a WGD event in the palm ancestor not shared with Dasypogonaceae may be responsible for this difference. It would be surprising to observe such a conspicuous pattern of chromosome number “doubling” given the propensity for idiosyncratic chromosomal number change post-WGD; such a doubling after the split of ancestral Arecaceae and Dasypogonaceae would have had to persist for >100 my of evolution (based on the divergence time estimates in Givnish et al. [2018]). However, just as palms display some of the slowest substitution rates among monocots (see Barrett et al. 2016), plant taxa with relatively longer generation times generally experience slower rates of postpolyploid diploidization, and perhaps the same is true for descending dysploidy (Mandáková and Lysak 2018).

Our finding of no significant relationship between genome size and chromosome number corroborates an earlier analysis based on comparison of genome size across different categories of chromosome numbers (Leitch et al. 2010). Changes in chromosome number can, however, be an important evolutionary force involved in species diversification, often following a polyploidy event. During post-WGD diploidization and fractionation, dysploid changes in chromosome number can result in reproductive isolation and thus cladogenesis (e.g., Dodsworth et al. 2016; Clark and Donoghue 2017; Mandáková and Lysak 2018). Although WGD (genome doubling or additive fusion) is an important factor in plant diversification in many clades, less study has been devoted to the evolutionary consequences of dysploidy, which appears to be the predominant mode of chromosomal evolution in palms. Additional sampling of both Dasypogonaceae and Arecaceae is needed, as is a more inclusive, phylogenetically comparative analysis of chromosome number across monocots, for example, including both anagenetic and cladogenetic changes (e.g., chromoSSE; Freyman and Höhna 2018).

## Conclusions and Future Directions

Here, we have unequivocally identified an ancient WGD event shared by all palms and characterized the predominant mode of chromosomal change in palms as dysploidy. Remaining questions include the role of repetitive elements in palm genome size evolution and how different genomic attributes have collectively influenced species diversification during the long evolutionary history of this ecologically dominant, evolutionarily successful clade. In the future, it will be critical to obtain whole-genome sequences for multiple representatives of each palm subfamily (including the genome of *N.fruticans*, the sole member of subfamily Nypoideae), along with each of the four genera of Dasypogonaceae. These genomic resources will allow 1) comparative analyses of genome architecture and synteny, 2) analysis of gene family expansion and contraction with respect to adaptive radiation of the palms, 3) ancestral reconstruction of genome content and architecture (i.e., gene family copy numbers, gene order along chromosomes, and repeat content), and 4) associations of genomic features, important phenotypic traits, ecology, biogeography, and species diversification rates. Such a densely sampled, integrative framework in the palms will advance our understanding of the evolution of tropical biodiversity.

## Supplementary Material

Supplementary data are available at *Genome Biology and Evolution* online.

## Supplementary Material

Supplementary_Material_evz092

## Notes

**Data deposition:** This project has been deposited at the NCBI Sequence Read Archive under the accession BioProject (PRJNA313089).

## References

[evz092-B1] Akaike H. 1974 A new look at the statistical model identification. IEEE Trans Automat Contr. 19(6):716–723.

[evz092-B2] Al-Mssallem IS, et al 2013 Genome sequence of the date palm *Phoenix dactylifera* L. Nat Commun. 4: 2274. 23917264 10.1038/ncomms3274 PMC3741641

[evz092-B3] Antonelli A, et al 2017 Toward a self-updating platform for estimating rates of speciation and migration, ages, and relationships of taxa. Syst Biol. 66(2):152–166. 27616324 10.1093/sysbio/syw066 PMC5410925

[evz092-B4] Asmussen CB, et al 2006 A new subfamily classification of the palm family (Arecaceae): evidence from plastid DNA phylogeny. Bot J Linn Soc. 151(1):15–38.

[evz092-B5] Bacon CD, Baker WJ, Simmons MP. 2012 Miocene dispersal drives island radiations in the palm tribe Trachycarpeae (Arecaceae). Syst Biol. 61(3):426–442. 22223444 10.1093/sysbio/syr123

[evz092-B6] Bacon CD, Velásquez-Puentes FJ, Hoorn C, Antonelli A. 2018 Iriarteeae palms tracked the uplift of Andean Cordilleras. J Biogeogr. 45(7):1653–1663.

[evz092-B7] Baker WJ, Couvreur T. 2013 Global biogeography and diversification of palms sheds light on the evolution of tropical lineages. I. Historical biogeography. J Biogeogr. 40(2):274–285.

[evz092-B8] Baker WJ, Dransfield J. 2016 Beyond genera palmarum: progress and prospects in palm systematics. Bot J Linn Soc. 182(2):207–233.

[evz092-B9] Balslev H, Bernal R, Fay MF. 2016 Palms—emblems of tropical forests. Bot J Linn Soc. 182(2):195–200.

[evz092-B10] Barrett CF, Davis JI, Leebens-Mack J, Conran JG, Stevenson DW. 2013 Plastid genomes and deep relationships among the commelinid monocot angiosperms. Cladistics 29(1):65–87. 10.1111/j.1096-0031.2012.00418.x 34814372

[evz092-B11] Barrett CF, et al 2016 Plastid genomes reveal support for deep phylogenetic relationships and extensive rate variation among palms and other commelinid monocots. New Phytol. 209(2):855–870. 26350789 10.1111/nph.13617

[evz092-B12] Bennett MD, Leitch IJ. 2012 Plant DNA C-values database release 6.0. Available from: http://data.kew.org/cvalues/ (accessed December 7, 2018).

[evz092-B13] Bennetzen JL, Ma JX, Devos K. 2005 Mechanisms of recent genome size variation in flowering plants. Ann Bot. 95(1):127–132. 15596462 10.1093/aob/mci008 PMC4246713

[evz092-B301] Birney E, Clamp M, Durbin R. GeneWise and Genomewise. Genome Res. 2004;14:988–995. 15123596 10.1101/gr.1865504 PMC479130

[evz092-B302] Bolger AM, Lohse M, Usadel B. Trimmomatic: a flexible trimmer for Illumina sequence data. Bioinformatics 2014;30:2114–2120. 24695404 10.1093/bioinformatics/btu170 PMC4103590

[evz092-B303] Buchfink B, Xie C, Huson DH. Fast and sensitive protein alignment using DIAMOND. Nat Methods. 2015;12:59–60. 25402007 10.1038/nmeth.3176

[evz092-B304] Cai J, et al The genome sequence of the orchid *Phalaenopsis equestris*. Nat Genet. 2015;47:65–72. 25420146 10.1038/ng.3149

[evz092-B305] Camacho C, et al BLAST+: architecture and applications. BMC Bioinformatics 2009;10:421. 20003500 10.1186/1471-2105-10-421 PMC2803857

[evz092-B306] Cámara-Leret R, Paniagua-Zambrana N, Svenning J-C, Balslev H, Macía MJ. Geospatial patterns in traditional knowledge serve in assessing intellectual property rights and benefit-sharing in northwest South America. J Ethnopharmacol. 2014;158:58–65. 25456422 10.1016/j.jep.2014.10.009

[evz092-B14] Cannon SB, et al 2015 Multiple polyploidy events in the early radiation of nodulating and nonnodulating legumes. Mol Biol Evol. 32(1):193–210. 25349287 10.1093/molbev/msu296 PMC4271530

[evz092-B307] Cavalier-Smith T. Nuclear volume control by nucleoskeletal DNA, selection for cell volume and cell growth rate, and the solution of the DNA C-Value Paradox. J Cell Sci. 1978;34:247–278. 372199 10.1242/jcs.34.1.247

[evz092-B15] Clark JW, Donoghue P. 2017 Constraining the timing of whole genome duplication in plant evolutionary history. Proc R Soc B Biol Sci. 284(1858):20170912. 10.1098/rspb.2017.0912 PMC5524505 28679730

[evz092-B16] Couvreur TL, Baker WJ. 2013 Tropical rain forest evolution: palms as a model group. BMC Biol. 11:48. 23587415 10.1186/1741-7007-11-48 PMC3627317

[evz092-B17] Couvreur TL, Forest F, Baker WJ. 2011 Origin and global diversification patterns of tropical rain forests: inferences from a complete genus-level phylogeny of palms. BMC Biol. 9:44. 21679405 10.1186/1741-7007-9-44 PMC3142250

[evz092-B18] Devos KM, Brown JKM, Bennetzen JL. 2002 Genome size reduction through illegitimate recombination counteracts genome expansion in *Arabidopsis*. Genome Res. 12:1075–1079. 12097344 10.1101/gr.132102 PMC186626

[evz092-B19] D’Hont A, et al 2012 The banana (*Musa acuminata*) genome and the evolution of monocotyledonous plants. Nature 488:213–217. 22801500 10.1038/nature11241

[evz092-B20] Dodsworth S, Chase MW, Leitch AR. 2016 Is post-polyploidization diploidization the key to the evolutionary success of angiosperms? Bot J Linn Soc. 180(1):1–5.

[evz092-B21] Dransfield J, et al 2008 Genera palmarum—the evolution and classification of palms. Richmond (United Kingdom): Royal Botanic Gardens, Kew 732 p.

[evz092-B22] Edger PP, et al 2015 The butterfly plant arms-race escalated by gene and genome duplications. Proc Natl Acad Sci U S A. 112(27):8362–8366. 26100883 10.1073/pnas.1503926112 PMC4500235

[evz092-B23] Eiserhardt WL, Svenning J-C, Baker WJ, Couvreur TLP, Balslev H. 2013 Dispersal and niche evolution jointly shape the geographic turnover of phylogenetic clades across continents. Sci Rep. 3:1164. 23383367 10.1038/srep01164 PMC3563030

[evz092-B24] Eiserhardt WL, Svenning J-C, Kissling WD, Balslev H. 2011 Geographical ecology of the palms (Arecaceae): determinants of diversity and distributions across spatial scales. Ann Bot. 108(8):1391–1416. 21712297 10.1093/aob/mcr146 PMC3219491

[evz092-B308] Emms DM, Kelly S. OrthoFinder: solving fundamental biases in whole genome comparisons dramatically improves orthogroup inference accuracy. Genome Biol. 2015;16:157. 26243257 10.1186/s13059-015-0721-2 PMC4531804

[evz092-B309] Estep MC, et al Allopolyploidy, diversification, and the Miocene grassland expansion. Proc Natl Acad Sci U S A. 2014;111:15149–15154. 25288748 10.1073/pnas.1404177111 PMC4210326

[evz092-B25] Faurby S, Eiserhardt WL, Baker WJ, Svenning J-C. 2016 An all-evidence species-level supertree for the palms (Arecaceae). Mol Phylogenet Evol. 100:57–69. 27060018 10.1016/j.ympev.2016.03.002

[evz092-B26] Felsenstein J. 1985 Phylogenies and the comparative method. Am Nat. 125(1):1–15.

[evz092-B27] Finnegan DJ. 1989 Eukaryotic transposable elements and genome evolution. Trends Genet. 5:103–107. 2543105 10.1016/0168-9525(89)90039-5

[evz092-B28] Fleischmann A, et al 2014 Evolution of genome size and chromosome number in the carnivorous plant genus Genlisea (Lentibulariaceae), with a new estimate of the minimum genome size in angiosperms. Ann Bot. 114(8):1651–1663. 25274549 10.1093/aob/mcu189 PMC4649684

[evz092-B29] Freyman WA, Höhna S. 2018 Cladogenetic and anagenetic models of chromosome number evolution: a Bayesian model averaging approach. Syst Biol. 67(2):195–215. 28945917 10.1093/sysbio/syx065

[evz092-B30] Garland T, Harvey PH, Ives AR. 1992 Procedures for the analysis of comparative data using phylogenetically independent contrasts. Syst Biol. 41(1):18–32.

[evz092-B31] Givnish TJ, et al 2010 Assembling the tree of the monocotyledons: plastome sequence phylogeny and evolution of Poales. Ann MO Bot Gard. 97(4):584–616.

[evz092-B32] Givnish TJ, et al 2018 Monocot plastid phylogenomics, timeline, net rates of species diversification, the power of multi-gene analyses, and a functional model for the origin of monocots. Am J Bot. 105(11):1888–1910. 30368769 10.1002/ajb2.1178

[evz092-B310] Glassman SF, 1999 A taxonomic treatment of the palm subtribe Attaleinae (Tribe cocoeae*)* Urbana (IL): University of Illinois Press.

[evz092-B33] Glick L, Mayrose I. 2014 ChromEvol: assessing the pattern of chromosome number evolution and the inference of polyploidy along a phylogeny. Mol Biol Evol. 31(7):1914–1922. 24710517 10.1093/molbev/msu122

[evz092-B311] Grabherr MG, et al Full-length transcriptome assembly from RNA-Seq data without a reference genome. Nat Biotechnol. 2011;29:644–652. 21572440 10.1038/nbt.1883 PMC3571712

[evz092-B312] Gregory TR. Coincidence, coevolution, or causation? DNA content, cell size, and the C-value enigma. Biol Rev. 2001;76:65–101. 11325054 10.1017/s1464793100005595

[evz092-B313] Gregory TR. Synergy between sequence and size in large-scale genomics. Nat Rev Genet. 2005;6:699–708. 16151375 10.1038/nrg1674

[evz092-B34] Grover CE, Wendel JF. 2010 Recent insights into mechanisms of genome size change in plants. J of Bot. 2010:1.

[evz092-B35] Gunn BF. 2004 The phylogeny of the Cocoeae (Arecaceae) with emphasis on *Cocos nucifera*. Ann Mo Bot Gard. 91:505–522.

[evz092-B36] Gunn BF, et al 2015 Ploidy and domestication are associated with genome size variation in Palms. Am J Bot. 102(10):1625–1633. 26437888 10.3732/ajb.1500164

[evz092-B314] Haas BJ, et al De novo transcript sequence reconstruction from RNA-seq using the Trinity platform for reference generation and analysis. Nat Protoc. 2013;8:1494–1512. 23845962 10.1038/nprot.2013.084 PMC3875132

[evz092-B315] Harkess A, et al The asparagus genome sheds light on the origin and evolution of a young Y chromosome. Nat Commun. 2017;8:1279. 29093472 10.1038/s41467-017-01064-8 PMC5665984

[evz092-B37] Hawkins JS, Grover CE, Wendel JF. 2008 Repeated big bangs and the expanding universe: directionality in plant genome size evolution. Plant Sci. 174(6):557–562.

[evz092-B38] He Z, et al 2015 De novo assembly of coding sequences of the mangrove palm (*Nypa fruticans*) using RNA-Seq and discovery of whole-genome duplications in the ancestor of palms. PLoS One 10(12):e0145385. 26684618 10.1371/journal.pone.0145385 PMC4684314

[evz092-B316] Johnson M. An unusually high chromosome number in *Voanioala gerardii* (Palmae: Arecoideae: Cocoeae: Butiinae) from Madagascar. Kew Bull. 1989;44:207.

[evz092-B39] Jiao Y, et al 2011 Ancestral polyploidy in seed plants and angiosperms. Nature 473(7345):97–100. 21478875 10.1038/nature09916

[evz092-B317] Katoh K, Standley DM. MAFFT multiple sequence alignment software version 7: improvements in performance and usability. Mol Biol Evol. 2013;30:772–780. 23329690 10.1093/molbev/mst010 PMC3603318

[evz092-B318] Kawahara Y, et al Improvement of the *Oryza sativa* Nipponbare reference genome using next generation sequence and optical map data. Rice 2013;6:4. 24280374 10.1186/1939-8433-6-4 PMC5395016

[evz092-B40] Kellogg EA. 2016 Has the connection between polyploidy and diversification actually been tested? Curr Op Plant Biol. 30:25–32. 10.1016/j.pbi.2016.01.002 26855304

[evz092-B41] Khabbazian M, Kriebel R, Rohe K, Ané C. 2016 Fast and accurate detection of evolutionary shifts in Ornstein–Uhlenbeck models. Methods Ecol Evol. 7(7):811–824.

[evz092-B42] Kissling WD, et al 2012 Quaternary and pre-Quaternary historical legacies in the global distribution of a major tropical plant lineage. Global Ecol Biogeogr. 21(9):909–921.

[evz092-B43] Kissling WD, et al 2012 Cenozoic imprints on the phylogenetic structure of palm species assemblages worldwide. Proc Natl Acad Sci U S A. 109(19):7379–7384. 22529387 10.1073/pnas.1120467109 PMC3358898

[evz092-B319] Kraaijeveld K. Genome size and species diversification. Evol Biol. 2010;37:227–233. 22140283 10.1007/s11692-010-9093-4 PMC3227167

[evz092-B320] Langmead B, Trapnell C, Pop M, Salzberg SL. Ultrafast and memory-efficient alignment of short DNA sequences to the human genome. Genome Biol. 2009;10:R25. 19261174 10.1186/gb-2009-10-3-r25 PMC2690996

[evz092-B44] Landis JB, et al 2018 Impact of whole-genome duplication events on diversification rates in angiosperms. Am J Bot. 105(3):348–363. 29719043 10.1002/ajb2.1060

[evz092-B45] Lavin M, Herendeen PS, Wojciechowski MF. 2005 Evolutionary rates analysis of Leguminosae implicates a rapid diversification of lineages during the tertiary. Syst Biol. 54(4):575–594. 16085576 10.1080/10635150590947131

[evz092-B46] Leitch IJ, Beaulieu JM, Chase MW, Leitch AR, Fay MF. 2010 Genome size dynamics and evolution in monocots. J Bot. 2010: 862516.

[evz092-B321] Li B, Dewey CN. RSEM: accurate transcript quantification from RNA-Seq data with or without a reference genome. BMC Bioinformatics 2011;12:323. 21816040 10.1186/1471-2105-12-323 PMC3163565

[evz092-B47] Mandáková T, Lysak MA. 2018 Post-polyploid diploidization and diversification through dysploid changes. Curr Opin Plant Biol. 42:55–65. 29567623 10.1016/j.pbi.2018.03.001

[evz092-B48] Matasci N, et al 2014 Data access for the 1,000 plants (1KP) project. GigaScience 3: 17. 25625010 10.1186/2047-217X-3-17 PMC4306014

[evz092-B49] McKain MR, et al 2016 A phylogenomic assessment of ancient polyploidy and genome evolution across the poales. Genome Biol Evol. 8(4):1150–1164. 26988252 10.1093/gbe/evw060 PMC4860692

[evz092-B322] Ming R, et al The pineapple genome and the evolution of CAM photosynthesis. Nat Genet. 2015;47:1435–1442. 26523774 10.1038/ng.3435 PMC4867222

[evz092-B323] Mirarab S, et al ASTRAL: genome-scale coalescent-based species tree estimation. Bioinformatics 2014;30:i541–i548. 25161245 10.1093/bioinformatics/btu462 PMC4147915

[evz092-B50] Panchy N, Lehti-Shiu MD, Shiu S-H. 2016 Evolution of gene duplication in plants. Plant Physiol. 171(4):2294–2316. 27288366 10.1104/pp.16.00523 PMC4972278

[evz092-B51] Paradis E, Schliep K. 2019 ape 5.0: an environment for modern phylogenetics and evolutionary analyses in R. Bioinformatics 35(3):526–528. 30016406 10.1093/bioinformatics/bty633

[evz092-B52] Paterson AH, et al 2009 Comparative genomics of grasses promises a bountiful harvest. Plant Physiol. 149(1):125–131. 19126703 10.1104/pp.108.129262 PMC2613718

[evz092-B53] Pellicer J, Fay MF, Leitch IJ. 2010 The largest eukaryotic genome of them all? Bot J Linn Soc. 164(1):10–15.

[evz092-B54] Pellicer J, Hidalgo O, Dodsworth S, Leitch IJ. 2018 Genome size diversity and its impact on the evolution of land plants. Genes 9:88. 10.3390/genes9020088 PMC5852584 29443885

[evz092-B55] Pfeil BE, Schlueter JA, Shoemaker RC, Doyle JJ. 2005 Placing paleopolyploidy in relation to taxon divergence: a phylogenetic analysis in legumes using 39 gene families. Syst Biol. 54(3):441–454. 16012110 10.1080/10635150590945359

[evz092-B56] Puttick MN, Clark J, Donoghue P. 2015 Size is not everything: rates of genome size evolution, not *C*-value, correlate with speciation in angiosperms. Proc R Soc B Biol Sci. 282(1820):20152289. 10.1098/rspb.2015.2289 PMC4685785 26631568

[evz092-B324] Ramírez-Rodríguez R, Tovar-Sánchez E, Jiménez Ramírez J, Vega Flores K, Rodríguez V. Introgressive hybridization between *Brahea dulcis* and *Brahea nitida* (Arecaceae) in Mexico: evidence from morphological and PCR–RAPD patterns. Botany 2011;89:545–557.

[evz092-B57] Ren R, et al 2018 Widespread whole genome duplications contribute to genome complexity and species diversity in angiosperms. Mol Plant 11(3):414–426. 29317285 10.1016/j.molp.2018.01.002

[evz092-B58] Renny-Byfield S, Wendel JF. 2014 Doubling down on genomes: polyploidy and crop plants. Am J Bot. 101:1711–1725. 25090999 10.3732/ajb.1400119

[evz092-B59] Revell LJ. 2012 phytools: an R package for phylogenetic comparative biology (and other things). Methods Ecol Evol. 3(2):217–223.

[evz092-B60] Röser M, Johnson MAT, Hanson L. 1997 Nuclear DNA amounts in palms (Arecaceae). Bot Acta. 110(1):79–89.

[evz092-B325] Röser M, 2000 DNA amounts and qualitative properties of nuclear genomes in palms (Arecaceae) In: Wilson KL, Morrison DA, editors. Monocots: systematics and evolution. Melbourne (VIC): CSIRO Publishing p. 538–544.

[evz092-B61] Schlueter JA, et al 2004 Mining EST databases to resolve evolutionary events in major crop species. Genome 47(5):868–876. 15499401 10.1139/g04-047

[evz092-B62] Singh R, et al 2013 Oil palm genome sequence reveals divergence of interfertile species in Old and New worlds. Nature 500(7462):335–339. 23883927 10.1038/nature12309 PMC3929164

[evz092-B63] Soltis DE, et al 2009 Polyploidy and angiosperm diversification. Am J Bot. 96(1):336–348. 21628192 10.3732/ajb.0800079

[evz092-B327] Stamatakis A. RAxML version 8: a tool for phylogenetic analysis and post-analysis of large phylogenies. Bioinformatics 2014;30:1312–1313. 24451623 10.1093/bioinformatics/btu033 PMC3998144

[evz092-B328] Suyama M, Torrents D, Bork P. PAL2NAL: robust conversion of protein sequence alignments into the corresponding codon alignments. Nucleic Acids Res. 2006;34:W609–W612. 16845082 10.1093/nar/gkl315 PMC1538804

[evz092-B64] Tang H, Bowers JE, Wang X, Paterson AH. 2010 Angiosperm genome comparisons reveal early polyploidy in the monocot lineage. Proc Natl Acad Sci U S A. 107(1):472–477. 19966307 10.1073/pnas.0908007107 PMC2806719

[evz092-B65] ter Steege HT, et al 2013 Hyperdominance in the Amazonian Tree Flora. Science 342:1243092. 24136971 10.1126/science.1243092

[evz092-B66] The Angiosperm Phylogeny Group. 2016 An update of the Angiosperm Phylogeny Group classification for the orders and families of flowering plants: aPG IV. Bot J Linn Soc. 181:1–20.

[evz092-B329] Thomas CA. The genetic organization of chromosomes. Annu Rev Genet. 1971;5:237–256. 16097657 10.1146/annurev.ge.05.120171.001321

[evz092-B67] Uhl NW, Dransfield J. 1987 Genera Palmarum, a classification of palms based on the work of Harold E. Moore, Jr. Lawrence (KS): L.H. Bailey Hortorium and the International Palm Society.

[evz092-B68] Unruh SA, et al 2018 Phylotranscriptomic analysis and genome evolution of the Cypripedioideae (Orchidaceae). Am J Bot. 105(4):631–640. 29608785 10.1002/ajb2.1047

[evz092-B69] van de Peer Y, Mizrachi E, Marchal K. 2017 The evolutionary significance of polyploidy. Nat Rev Genet. 18(7):411–424. 28502977 10.1038/nrg.2017.26

[evz092-B70] Wagenmakers E-J, Farrell S. 2004 AIC model selection using Akaike Weights. Psychon Bull Rev. 11:192–196. 15117008 10.3758/bf03206482

[evz092-B71] Wendel JF. 2015 The wondrous cycles of polyploidy in plants. Am J Bot. 102(11):1753–1756. 26451037 10.3732/ajb.1500320

[evz092-B72] Wood TE, et al 2009 The frequency of polyploid speciation in vascular plants. Proc Natl Acad Sci U S A. 106(33):13875–13879. 19667210 10.1073/pnas.0811575106 PMC2728988

[evz092-B73] Zenil-Ferguson R, Ponciano JM, Burleigh JG. 2016 Evaluating the role of genome downsizing and size thresholds from genome size distributions in angiosperms. Am J Bot. 103(7):1175–1186. 27206462 10.3732/ajb.1500408

[evz092-B330] Zhang J. Evolution by gene duplication: an update. Trends Ecol Evol. 2003;18:292–298.

[evz092-B74] Zhang G-Q, et al 2017 The Apostasia genome and the evolution of orchids. Nature 549(7672):379–383. 28902843 10.1038/nature23897 PMC7416622

