# Supplementary material for: Ancient Polyploidy and Genome Evolution in Palms
Source: Genome Biol Evol. 2019 Apr 27;11(5):1501–11. doi: 10.1093/gbe/evz092 (PMC6535811; doi:10.1093/gbe/evz092)
Supplement: Supplementary_Material_evz092 [file supplementary_material_evz092.docx]

SUPPLEMENTARY INFORMATION

**Table S1**. A summary of RNA-seq and genome skim datasets used in this study. Herbarium codes: FTG = Fairchild Tropical Botanical Garden; GA = University of Georgia; NY = New York Botanical Garden; PERTH = Western Australian Herbarium

| Taxon | Transcripts | Median Length | Average Length | Total Bases | Source |
| --- | --- | --- | --- | --- | --- |
| *Acorus americanus* | 86592 | 482 | 766 | 66368105 | 1KP |
| *Baxteria australis* | 84888 | 313 | 401 | 34078246 | Conran 906 (PERTH) |
| *Calectasia_grandiflora* | 9699 | 252 | 290 | 2818028 | Thiele & Conran 928 (PERTH) |
| *Chamaedorea seifrizii* | 119187 | 422 | 655 | 78166307 | Zomlefer et al. 2358 (GA) |
| *Cocos nucifera* | 85332 | 493 | 920 | 78519240 | 1KP |
| *Costus pulverulentus* | 124418 | 333 | 471 | 58672158 | Zomlefer et al. 2294 (FTG, NY) |
| *Dasypogon bromeliifolius* | 254910 | 452 | 784 | 199909426 | Thiele & Conran 921 (PERTH) |
| *Hanguana malayana* | 144326 | 404 | 643 | 92852509 | Stevenson 0039-0 (NY) |
| *Howea belmoreana* | 160346 | 438 | 777 | 124621642 | SRA |
| *Howea forsteriana* | 122857 | 530 | 891 | 109482803 | SRA |
| *Kingia australis* | 132870 | 303 | 392 | 52185512 | Thiele & Conran 992 (PERTH) |
| *Mauritia flexosa* | 249993 | 398 | 735 | 183970008 | SRA |
| *Neoregelia carolinae* | 119685 | 448 | 743 | 88945556 | SRA |
| *Nypa fruticans* | 124040 | 491 | 840 | 104254580 | 1KP |
| *Sabal bermundana* | 130625 | 382 | 613 | 80151546 | 1KP |
| *Serenoa repens* | 152514 | 324 | 477 | 72874783 | 1KP |
| *Tradescantia paludosa* | 57137 | 318 | 435 | 24889096 | Stevenson 82-221 (NY) |
| *Typha latifolia* | 125456 | 526 | 864 | 108418288 | 1KP |

**Table S2**. Chromosome numbers (2n) and genome sizes (1C in picograms and Gigabases). Blue text indicates taxa used for ancestral state reconstruction of genome size. * = a different species used in genome size analysis; ** = not included in chromosome number analysis. Data are from the Kew Angiosperm C-value database (Bennett and Leitch, 2014) and Genera Palmarum II (Dransfield, Uhl, et al., 2008).

| Species | 1C | Gigabases | Chromosome # (2n) |
| --- | --- | --- | --- |
| *Acrocomia aculeata* | 3.39 | 3.31 | 30 |
| *Acrocomia crispa* |  |  | 30 |
| *Adonidia merrillii* |  |  | 32 |
| *Aiphanes minima* |  |  | 30 |
| *Allagoptera arenaria* |  |  | 32 |
| *Allagoptera caudescens* |  |  | 32 |
| *Archontophoenix alexandrae* |  |  | 32 |
| *Archontophoenix cunninghamiana* | 4.74 | 4.64 | 32 |
| *Areca catechu* |  |  | 32 |
| *Areca concinna* | 6.01 | 5.88 | 32 |
| *Areca macrocalyx* |  |  | 32 |
| *Areca triandra* |  |  | 32 |
| *Arenga caudata* |  |  | 64 |
| *Arenga engleri* |  |  | 32 |
| *Arenga obtusifolia* |  |  | 32 |
| *Arenga pinnata* |  |  | 32 |
| *Arenga porphyrocarpa* |  |  | 32 |
| *Arenga tremula* |  |  | 32 |
| *Arenga undulatifolia* |  |  | 32 |
| *Arenga wightii* |  |  | 32 |
| *Astrocaryum mexicanum* |  |  | 30 |
| *Attalea allenii* |  |  | 32 |
| *Attalea cohune* |  |  | 32 |
| *Attalea eichleri* |  |  | 32 |
| *Attalea speciosa* |  |  | 32 |
| *Bactris gasipaes* |  |  | 30 |
| **Bactris hondurensis* | 4.07 | 3.98 | 30 |
| *Beccariophoenix madagascariensis* | 1.80 | 1.76 | 36 |
| *Bentinckia condapanna* | 2.78 | 2.72 | 32 |
| *Bentinckia nicobarica* |  |  | 32 |
| ***Bismarckia nobilis* | 2.03 | 1.98 | 36 |
| *Borassus flabellifer* | 8.60 | 8.41 | 36 |
| *Brahea aculeata* |  |  | 36 |
| *Brahea armata* |  |  | 36 |
| *Brahea dulcis* | 1.06 | 1.03 | 36 |
| *Brassiophoenix schumannii* |  |  | 32 |
| *Butia capitata* |  |  | 32 |
| *Calamus caesius* |  |  | 26 |
| *Calamus caryotoides* | 1.17 | 1.14 | 26 |
| *Calamus ciliaris* |  |  | 26 |
| *Calamus erectus* |  |  | 26 |
| *Calamus guruba* |  |  | 26 |
| *Calamus longisetus* |  |  | 26 |
| *Calamus ornatus* |  |  | 26 |
| *Calamus rotang* |  |  | 26 |
| *Calamus viminalis* |  |  | 26 |
| *Calyptrocalyx forbesii* |  |  | 32 |
| *Calyptrogyne ghiesbreghtiana* | 3.41 | 3.34 | 28 |
| *Calyptronoma occidentalis* |  |  | 28 |
| *Calyptronoma plumeriana* |  |  | 28 |
| *Calyptronoma rivalis* |  |  | 28 |
| *Caryota rumphiana* |  |  | 34 |
| *Caryota urens* | 6.61 | 6.46 | 34 |
| *Ceratolobus concolor* |  |  | 26 |
| *Ceratolobus pseudoconcolor* |  |  | 26 |
| *Ceroxylon alpinum* |  |  | 36 |
| *Ceroxylon parvifrons* |  |  | 36 |
| *Ceroxylon* sp*.* | 3.86 | 3.77 | 36 |
| *Chamaedorea alternans* |  |  | 32 |
| *Chamaedorea arenbergiana* |  |  | 32 |
| *Chamaedorea brachypoda* |  |  | 26 |
| *Chamaedorea cataractarum* |  |  | 26 |
| *Chamaedorea elatior* |  |  | 26 |
| *Chamaedorea elegans* |  |  | 26 |
| *Chamaedorea ernesti-augusti* |  |  | 26 |
| *Chamaedorea glaucifolia* |  |  | 26 |
| *Chamaedorea klotzschiana* | 4.09 | 3.99 | 26 |
| *Chamaedorea microspadix* |  |  | 26 |
| *Chamaedorea oblongata* | 4.20 | 4.11 | 26 |
| *Chamaedorea parvisecta* |  |  | 26 |
| *Chamaedorea pinnatifrons* | 4.45 | 4.35 | 26 |
| *Chamaedorea pochutlensis* |  |  | 26 |
| *Chamaedorea pumila* |  |  | 26 |
| *Chamaedorea radicalis* |  |  | 26 |
| *Chamaedorea sartorii* |  |  | 26 |
| *Chamaedorea schiedeana* |  |  | 26 |
| *Chamaedorea seifrizii* | 2.91 | 2.84 | 26 |
| *Chamaedorea tepejilote* |  |  | 32 |
| *Chambreyronia macrocrapa* |  |  | 32 |
| *Chuniophoenix nana* | 1.54 | 1.50 | 36 |
| *Clinostigma exorrhizum* |  |  | 32 |
| *Clinostigma savoryanum* |  |  | 32 |
| *Coccothrinax argentata* | 7.44 | 7.27 | 36 |
| *Coccothrinax barbadensis* |  |  | 36 |
| *Coccothrinax crinita* |  |  | 36 |
| *Coccothrinax fragrans* | 6.48 | 6.34 | 36 |
| *Coccothrinax inaguensis* |  |  | 36 |
| *Coccothrinax littoralis* |  |  | 36 |
| *Coccothrinax miraguama* |  |  | 36 |
| *Cocos nucifera* | 3.55 | 3.47 | 32 |
| *Copernicia alba* |  |  | 36 |
| *Copernicia baileyana* |  |  | 36 |
| *Copernicia glabrescens* |  |  | 36 |
| *Copernicia hospita* |  |  | 36 |
| *Copernicia macroglossa* |  |  | 36 |
| *Copernicia prunifera* |  |  | 36 |
| *Copernicia rigida* |  |  | 36 |
| *Copernicia yarey* |  |  | 36 |
| *Corypha umbraculifera* |  |  | 36 |
| *Corypha utan* |  |  | 36 |
| *Cryosophila stauracantha* |  |  | 36 |
| *Cyphosperma trichospadix* |  |  | 32 |
| *Cyrtostachys renda* |  |  | 32 |
| ***Daemonorops verticillaris* | 2.78 | 2.71 | 26 |
| *Desmoncus polyacanthos* | 2.99 | 2.93 | 30 |
| *Drymophloeus litigiosus* |  |  | 32 |
| *Dypsis pilulifera* | 2.54 | 2.48 | 32 |
| *Dypsis scottiana* | 2.47 | 2.41 | 32 |
| *Elaeis guineensis* | 1.0 | 0.98 | 32 |
| *Elaeis oleifera* |  |  | 32 |
| *Euterpe oleracea* |  |  | 36 |
| *Euterpe precatoria* | 5.31 | 5.19 | 36 |
| *Gaussia attenuata* |  |  | 28 |
| *Geonoma camana* |  |  | 28 |
| *Geonoma interrupta* | 3.63 | 3.55 | 28 |
| *Guihaia argyrata* | 5.94 | 5.81 | 36 |
| *Heterospathe elata* |  |  | 32 |
| *Howea forsteriana* | 3.43 | 3.35 | 32 |
| *Hydriastele beguinii* |  |  | 32 |
| *Hydriastele costata* |  |  | 32 |
| *Hydriastele hombronii* |  |  | 32 |
| *Hyophorbe lagenicaulis* |  |  | 32 |
| *Hyphaene coriacea* |  |  | 36 |
| *Hyphaene dichotoma* |  |  | 36 |
| **Hyphaene petersiana* | 3.4 | 3.33 |  |
| *Hyphaene thebaica* |  |  | 36 |
| ***Iriartea deltoidea* | 12.28 | 12.01 | 32 |
| *Johannesteijsmannia altifrons* | 1.62 | 1.59 | 34 |
| ***Jubea chilensis* | 2.55 | 2.49 | 32 |
| *Kentiopsis oliviformis* |  |  | 32 |
| *Latania loddigesii* |  |  | 28 |
| *Latania lontaroides* | 3.5 | 3.42 | 28 |
| *Latania verschaffeltii* |  |  | 28 |
| ***Lepidocaryum tenue* | 4.10 | 4.01 | 30 |
| *Licuala peltata* |  |  | 28 |
| *Licuala spinosa* |  |  | 28 |
| *Linospadix minor* |  |  | 32 |
| *Livistona australis* |  |  | 36 |
| *Livistona chinensis* |  |  | 36 |
| ***Loxococcus rupicola* | 3.58 | 3.50 | 32 |
| *Marojejya darianii* |  |  | 32 |
| *Masoala madagascariensis* | 2.68 | 2.62 | 32 |
| *Mauritia flexuosa* | 4.72 | 4.62 | 30 |
| *Medemia argun* | 3.63 | 3.55 |  |
| *Metroxylon sagu* |  |  | 26 |
| *Neonicholsonia watsonii* |  |  | 36 |
| ***Normanbya normanbyi* | 4.86 | 4.75 |  |
| ***Nypa fruticans* | 1.19 | 1.16 |  |
| *Oenocarpus bataua* | 3.93 | 3.84 | 36 |
| *Phoenix dactylifera* | 0.95 | 0.93 | 36 |
| *Phoenix canariensis* |  |  | 36 |
| *Phoenix loureiroi* |  |  | 36 |
| *Phoenix paludosa* |  |  | 36 |
| *Phoenix pusilla* |  |  | 36 |
| *Phoenix reclinata* |  |  | 36 |
| *Phoenix roebelenii* | 1.53 | 1.49 | 36 |
| *Phoenix rupicola* | 1.50 | 1.47 | 36 |
| *Phoenix sylvestris* |  |  | 36 |
| *Phoenix theophrasti* | 1.32 | 1.29 | 36 |
| *Physokentia dennisii* |  |  | 32 |
| *Phytelephas aequatorialis* | 0.97 | 0.95 | 36 |
| *Phytelephas macrocarpa* | 0.998 | 0.98 | 36 |
| *Pigafetta filaris* |  |  | 28 |
| *Pinanga coronata* | 8.86 | 8.66 | 32 |
| *Prestoea decurrens* |  |  | 36 |
| *Pritchardia pacifica* |  |  | 36 |
| *Pritchardia thurstonii* |  |  | 36 |
| *Pseudophoenix sargentii* | 2.83 | 2.77 | 34 |
| *Pseudophoenix vinifera* |  |  | 34 |
| *Ptychosperma elegans* |  |  | 32 |
| *Ptychosperma macarthurii* |  |  | 32 |
| *Ptychosperma sanderianum* |  |  | 32 |
| *Ravenea glauca* | 2.31 | 2.25 | 32 |
| *Ravenea musicalis* | 3.03 | 2.96 | 32 |
| *Rhapis excelsa* | 4.80 | 4.69 | 36 |
| *Rhapis subtilis* |  |  | 36 |
| *Rhopalo­blaste ceramica* |  |  | 32 |
| *Rhopalostylis baueri* |  |  | 32 |
| *Roystonea oleracea* |  |  | 36 |
| *Roystonea princeps* |  |  | 36 |
| *Roystonea regia* |  |  | 36 |
| *Sabal bermudana* |  |  | 36 |
| *Sabal causiarum* |  |  | 36 |
| *Sabal gretherae* |  |  | 36 |
| *Sabal maritima* |  |  | 36 |
| *Sabal mauritiiformis* | 2.26 | 2.21 | 36 |
| *Sabal mexicana* |  |  | 36 |
| *Sabal minor* |  |  | 36 |
| *Sabal palmetto* |  |  | 36 |
| *Sabal pumos* |  |  | 36 |
| *Sabal uresana* |  |  | 36 |
| *Sabal yapa* |  |  | 36 |
| *Salacca affinis* |  |  | 28 |
| *Salacca glabrescens* |  |  | 28 |
| *Salacca zalacca* | 1.30 | 1.27 | 28 |
| ***Socratea exorrhiza* | 4.56 | 4.45 | 36 |
| ***Sommieria leucophylla* | 5.79 | 5.67 | 34 |
| *Syagrus amara* |  |  | 32 |
| *Syagrus comosa* |  |  | 32 |
| *Syagrus glaucescens* | 3.44 | 3.36 | 32 |
| *Syagrus orinocensis* |  |  | 32 |
| *Syagrus romanzoffiana* | 3.05 | 2.98 | 32 |
| *Syagrus schizophylla* |  |  | 32 |
| *Thrinax excelsa* |  |  | 36 |
| *Thrinax parviflora* |  |  | 36 |
| *Thrinax radiata* |  |  | 36 |
| *Trachycarpus fortunei* |  |  | 36 |
| *Trachycarpus martianus* |  |  | 36 |
| *Trachycarpus nanus* | 5.55 | 5.42 | 36 |
| *Trithrinax brasiliensis* |  |  | 36 |
| *Trithrinax campestris* |  |  | 36 |
| ***Voanioala gerardii* | 39.10 | 38.24 | >550, >596 |
| *Washingtonia filifera* | 1.55 | 1.52 | 36 |
| *Wendlandiella gracilis* | 2.86 | 2.80 | 28 |

**Table S3**. Summary of unique and total duplications (paralogs) per selected clades corresponding to Fig. 1.

| WGD Event | Unique Duplications  with 80% Bootstrap | Unique Duplications with 50% Bootstrap | All Paralog Pairs  with 80% Bootstrap | All Paralog Pairs with 50% Bootstrap |
| --- | --- | --- | --- | --- |
| Areceae + Cocoseae | 94 | 112 | 285 | 350 |
| Arecaceae | 278 | 300 | 3321 | 3573 |
| *Ananas* + *Neoregelia* | 196 | 253 | 740 | 872 |
| Commelinids | 108 | 247 | 2705 | 4822 |
| Tau | 731 | 776 | 23975 | 24888 |
| Sigma | 228 | 308 | 902 | 1255 |
| *Musa* + *Costus* | 538 | 656 | 2011 | 2775 |
| Commelinales + Zingiberales | 283 | 405 | 2663 | 3656 |

**Table S4**. Results from ChromEvol, in which explicit models of chromosome number evolution were compared. ‘AIC’ = Akaike information criterion; ‘AICw’ = Akaike weight; ‘λ, λ_1_’ = ascending dysploidy parameters; ‘δ, δ_1_’ = descending dysploidy parameters; ‘ρ’ = WGD parameter; ‘μ’ = demi-polyploidy parameter; ‘ν, β’ = base number and rate parameters.

|  |  |  |  | Parameter estimates | | | | | | | |
| --- | --- | --- | --- | --- | --- | --- | --- | --- | --- | --- | --- |
|  | Model | AIC | AICw | λ | λ_1_ | δ | δ_1_ | ρ | μ | ν | β |
| 1 | Constant rate | 711.4 | 0.216 | 24.6 | - | 19.3 | - | 0.14 | - | - | - |
| 2 | Constant rate + demi-polyploidy | 711.4 | 0.216 | 25.4 | - | 19.0 | - | 0.12 | - | - | - |
| 3 | Constant rate + demi-polyploidy (est) | 713.3 | 0.084 | 24.8 | - | 19.8 | - | 0.14 | 0.05 | - | - |
| 4 | Constant rate, no WGD | 759.0 | 0.000 | 45.3 | - | 11.9 | - | - | - | - | - |
| 5 | Linear rate | 711.0 | 0.264 | 33.8 | -0.5 | 0.07 | 1.39 | 0.15 | - | - | - |
| 6 | Linear rate + demi-polyploidy | 714.2 | 0.053 | 28.2 | -0.4 | 23.0 | -0.15 | 0.14 | - | - | - |
| 7 | Linear rate + demi-polyploidy (est) | 714.3 | 0.051 | 44.3 | -0.6 | 0 | 1.15 | 0.14 | 0 | - | - |
| 8 | Linear rate, no WGD | 756.9 | 0.000 | 10.9 | 2.1 | 0 | 0.81 | - | - | - | - |
| 9 | Chromosome changes by base # | 713.2 | 0.088 | 26.2 | - | 17.5 | - | 0.11 | - | 0.03 | 16 |
| 10 | Chromosome changes by base #, no WGD | 715.4 | 0.029 | 25.1 | - | 18.8 | - | - | - | 0.156 | 16 |

**Fig. S1**. Representation of phylogenetic (Antonelli et al., 2017), genome size, chromosome, and genome skim datasets.





**Fig. S2**. Maximum likelihood ancestral state reconstructions of chromosome number under various models in the palms. **A**. Chromosome number under Brownian Motion (BM). ‘2n = 32’ is the ancestral chromosome number estimate for the palms under BM. **B**. Estimation of rate shifts in chromosome number under an OU model. ‘*’ = significant shifts in trait values, also denoted by different colors.
